# Empirical identification and validation of tumor-targeting T cell receptors from circulation using autologous pancreatic tumor organoids

**DOI:** 10.1136/jitc-2021-003213

**Published:** 2021-11-16

**Authors:** Qingda Meng, Shanshan Xie, G Kenneth Gray, Mohammad H Dezfulian, Omar Gandarilla, Weilin Li, Ling Huang, Dipikaa Akshinthala, Elizabeth Ferrer, Catherine Conahan, Sofia Perea Del Pino, Joseph Grossman, Stephen J Elledge, Manuel Hidalgo, Senthil K Muthuswamy

**Affiliations:** 1 Department of Medicine, Beth Israel Deaconess Medical Center, Harvard Medical School, Boston, Massachusetts, USA; 2 Department of Cell Biology, Harvard Medical School, Boston, Massachusetts, USA; 3 Department of Genetics, Harvard Medical School, Boston, Massachusetts, USA; 4 Division of Hematology and Medical Oncology, Weill Cornell Medicine, New York, New York, USA

**Keywords:** clonal selection, antigen-mediated, gastrointestinal neoplasms, immunologic techniques

## Abstract

**Background:**

Tumor-specific cytotoxic T cells and T cell receptors are effective tools for cancer immunotherapy. Most efforts to identify them rely on known antigens or lymphocytes that have infiltrated into the tumor bed. Approaches to empirically identify tumor-targeting T cells and T cell receptors by exploiting all antigens expressed on tumor cell surfaces are not well developed for most carcinomas, including pancreatic cancer.

**Methods:**

Autologous tumor organoids were stimulated with T cells from the patients’ peripheral blood for 2 weeks to generate the organoid-primed T (opT) cells. opT cell phenotype was analyzed by monitoring changes in the expression levels of 28 cell surface and checkpoint proteins. Expression of ligands of the immune checkpoints was investigated by immunohistochemistry staining. T cells were labeled with carboxyfluorescein succinimidyl ester (CFSE) and assayed by flow cytometry to monitor tumor-induced T cell proliferation changes. opT cell-mediated killing of three-dimensional organoids was measured using an M30 ELISA kit. T cell receptors (TCRs) were identified by deep sequencing of gDNA isolated from T cells, and the TCR specificity was confirmed by transferring TCRs to the T cell line SKW-3 or donor T cells.

**Results:**

The co-culture was effective in the generation of CD8 + or CD4+opT cells. The opT cells killed autologous tumors in a granzyme B or Fas-Fas ligand-dependent manner and expressed markers of tissue-resident memory phenotype. Each patient-derived opT cell culture displayed a unique complement of checkpoint proteins. Interestingly, only NKG2A blockade showed a potent increase in the interferon-γ production compared with blocking programmed cell death protein 1 (PD-1) or programmed cell death ligand 1 (PD-L1) or TIM3 or TIGIT or LAG3. Importantly, TCR sequencing demonstrated a dramatic clonal expansion of T cells with a restricted subset of TCRs. Cloning and transferring the TCRs to heterologous T cells was sufficient to confer tumor cell recognition and cytotoxic properties in a patient-specific manner.

**Conclusion:**

We report a platform for expanding tumor-targeting T cells from the peripheral blood of patients with pancreatic cancer. We identify the NKG2A-HLA-E axis as a potentially important checkpoint for CD8 +T cells for pancreatic cancer. Lastly, we demonstrate empirical identification of tumor-targeting TCRs that can be used for TCR-therapeutics.

## Introduction

Identifying T cells with specific reactivity against tumor cells and the receptors they express will significantly advance cancer immunotherapy. Tumor-infiltrating lymphocytes (TILs), especially the cytotoxic CD8 +T cells used for adoptive immunotherapy^1–4^ are excellent for the identification of tumor-targeting T cell receptors (TCRs). TCRs expressed in TILs show a broad clonal expansion ranging from 0.1%–50%.^5 6^ However, the limited TIL populations in many carcinomas (including the prostate, estrogen receptor+breast, and pancreatic cancer) and the intratumor heterogeneity for TIL populations,^7–9^ creates challenges for obtaining T cells to identify tumor-targeting TCRs, which can be harnessed for cancer immunotherapy.

In contrast to TILs, the TCR repertoire in the blood is more diverse, with a wide range of epitope specificities. Previous reports have demonstrated expansion of tumor-targeting T cell populations by culturing peripheral blood-derived T cells in the presence of either tumor-associated antigens or antibodies targeting activated T cell markers such as 41BB and programmed cell death protein 1 (PD-1).^10–12^ Although effective, these approaches are usually targeted towards known tumor-associated antigens and do not exploit the diversity of antigens expressed on the tumor cell surface. For example, almost all current TCR-therapy efforts use TCRs targeting known antigens such as NY-ESO-1, which is not effective in many cancers, including pancreatic.^13^

Co-culture of epithelial tumor cell lines with autologous peripheral blood mononuclear cells (PBMCs) can generate tumor-specific T cells.^14^ Patient tumor-derived organoids preserve the histology, gene mutations, and complex epithelial lineages of the tumor tissue.^15–17^ They are a better representative of tumor antigens than established cell lines that suffer from genetic and phenotypic drift during long-term cell culture. Tumor organoids co-cultured with peripheral blood lymphocytes can generate populations of cytotoxic T cells.^18 19^ However, it is still unknown whether organoids can be used to induce clonal expansion of tumor-targeting T cells from peripheral blood and whether the expanded T cells express TCRs that can confer cytotoxic abilities against tumor cells when expressed in heterologous T cells. Here we report the achievement of a high degree of clonal expansion of memory T cells from PBMCs and empirical identification of tumor-targeting TCRs. We further demonstrate that the identified TCRs are sufficient to elicit patient-specific tumor recognition and killing when expressed in allogeneic T cells.

## Materials and methods

### Patient samples and organoids culture

Peripheral blood and tumor tissue were obtained from patients with a confirmed diagnosis of metastatic pancreatic cancer. Organoids were cultured as previously described.^16^ Culture media was replaced every 4 days. The human organoids for all experiments were between passages 7 and 12.

### Generation of human pancreatic tumor organoid-primed T cells and tumor-infiltrating lymphocytes

The PBMC fraction was isolated from peripheral blood by Ficoll-Paque density. PBMCs were cultured for 10 days with human T cell medium (HTM): Serum-free medium (CellGernix, 20801–0100), 10% human AB serum (Innovative Research, IPLA-SerAB-13458), human IL-2 (1000 IU/mL, Prospec, cyt-209), human IL-15 (10 ng/mL, Prospec, cyt-230), human IL-21 (10 ng/mL, Prospec, cyt-408), 1% penicillin–streptomycin (Gibco, 15140–122), 1% amphotericin B solution (Sigma, A2942) and ciprofloxacin (Fisher Scientific, 50255729). After 10 days of culture, 100,000 PBMCs were co-cultured with autologous tumor organoids containing 100,000 cells (see online supplemental methods for further details) per well, three wells per condition, in a 96 well flat bottom plate (Falcon, 353072) in HTM. On day 7, the cells were transferred to a new 24 well plate (Falcon, 353047), and the PBMCs were stimulated again with autologous organoids at a ratio of 1:1 for additional 7 days. Tumor-infiltrating lymphocyte (TIL) generation was performed as previously described using HTM.^20^

10.1136/jitc-2021-003213.supp1 Supplementary data

### Live imaging, 3D killing assay and IFN-γ and granzyme B production

The organoids were labeled with Green Dye (Thermo Fisher, C7025), and organoid-primed T (opT) cells were added at a ratio of 1:10 (Tumor cell: T cell). Images were taken every 10 min starting at 2.0-hour co-culture for a total of 20 hours. For killing assay, opT cells were added to three-dimensional (3D) organoids at a ratio of 1:1 and incubated at 37°C for 24 hours, 48 hours, and 72 hours, respectively. Supernatants were collected and used for ELISA: M30 Apoptosense CK18 Kit (DiaPharma, P10011), interferon (IFN)-γ ELISA kit (Mabtech, 3420–1 H-20), and granzyme B ELISA kit (Mabtech, 3485–1 H-20).

### FasL expression and opT 3D killing block with FasL antibody

Fas-Fas ligand (FasL) expression in opT cells from Patient 3 and Patient 38 was tested by immunoblot (FasL antibody, Biolegend#306409; β-actin antibody, Sigma#A5441) or flow cytometry (FasL-PE, Biolegend#306406). Patient 3 opT and Patient 38 opT cells were co-cultured with autologous tumor organoids as outlined above in the 3D killing assay in the presence of 1.0 µg/mL anti-FasL antibody (Biolegend, 306409). Supernatants were harvested after 72 hours and tested by M30 Apoptosense CK18 Kit (DiaPharma, P10011).

### Phenotype and memory marker test by CyTOF

Twenty-eight mass-labeled cell surface antibodies were used (details of information were included in online supplemental table S1C). Cell staining was performed as Maxpar Cell Surface Staining with Fresh Fix (Fluidigm) and analyzed using Helios at Dana-Farber mass cytometry core.

10.1136/jitc-2021-003213.supp2 Supplementary data

10.1136/jitc-2021-003213.supp3 Supplementary data

### TCRα, β and δ chain sequencing, TCR construction, transduction and SKW-3 activation

Genomic DNA was used for TCR sequencing by Adaptive Biotechnologies. Total productive TCR reads per sample varied between 157,295 to 283,448. The data were analyzed with Immanalyses V.3.0 (ImmunoSEQ).

#### TCR construction

TCRα–TCRβ chains were matched on the basis of representation observed in TCR sequencing reactions. Full-length TCR-encoding sequences were constructed by fusing the CDR3 region from opT TCR (provided by ImmunoSEQ sequencing analysis) with the missing conserved V-region and J-region-encoding sequences obtained from the international immunogenetics information system (IMGT) database. The human constant region was replaced with mouse C region, and P2A was added between the β chain and α chain.

#### TCR transduction, infection, and purification

HEK293T cells were transfected with TCR encoding pHAGE plasmid(s), the viral supernatants were collected and used to infect SKW-3 cells in the presence of polybrene (8 µg/mL). After 3 days of infection, infected cells were selected using puromycin (1 µg/mL). TCR-positive cells were sorted by FACS using mTCR antibody (Biolegend, 109212). SKW-3 cells were also transduced with human CD8 αβ and selected using blasticidin (final 10 µg/mL) after infection. Organoids in a 3D structure were co-cultured with SKW-3 cells for 16 hours, and changes in levels of CD69, an activation marker, was analyzed by flow cytometry.

### TCR transfer to allogeneic CD8+ T cells and tumor recognition

Donor PBMC-derived CD8 +T cells were transfected with the chimeric TCR vector and sorted using a mouse TCR antibody. Organoids in the 3D structure were co-cultured with TCR transferred T cells, and supernatants were collected and tested for IFN-γ secretion by ELISA (Mabtech, 3420–1 H-20). For MHC blocking experiment, purified, Pan-MHC Class I antibody (clone W6/32, Biolegend, 311428), Pan-MHC Class II antibody (clone Tu39, BD Pharmingen, 555556) antibodies were used at 10 µg/mL.

### Expression and blocking of checkpoint proteins

PD-1 and cytotoxic T-lymphocyte associated protein 4 (CTLA-4) were tested by cytometry by time-of-flight (CyTOF) (antibody details in online supplemental table S1), and the others were analyzed using antibodies in online supplemental table S3 by flow cytometry. Immune regulation markers include TIM3, LAG3, TIGIT, GITR, BTLA, CD96, NKG2A, KIR2DL1/DL2/DL3/DL4/DL5, and KIR3DL1/DL2/DL3. Organoids in the 3D structure were incubated with opT cells in the presence of 10 µg/mL antibody or 2 µg/mL soluble protein: PD-1 (Selleckchem, A2002), PD-L1 (Biolegend, 329716), TIM3 (Biolegend, 345038); recombinant protein NKG2A (Prospec, PRO-2440), TIM3 (Prospec, HAV-221), TIGIT (Prospec, PRO-2498) and LAG3 (R&D, 2319-L3-050). The supernatants were analyzed for IFN-γ using an ELISA kit (Mabtech, 3420–1 H-20).

10.1136/jitc-2021-003213.supp5 Supplementary data

### Generation of mouse organoids and mouse opT cells and killing assay

Mouse organoids were generated from triple-negative breast cancer mouse models.^21^ Autologous mouse T cells from the spleen were isolated by EasySep Mouse T Cell Isolation Kit (Stemcell Technologies, 19851). To generate opT, mouse organoids, and T cells were cultured in mouse T cell medium: Serum-free medium (CellGernix, 20 801–0100), 10% fetal bovine serum (FBS) (Glico, 16140071), mouse interleukin (IL)-2 (1000 IU/mL) (Peprotech, 400–02), mouse IL-15 (10 ng/mL, Peprotech, 400–24) and mouse IL-21 (10 ng/mL, Prospec, CYT-033). For killing assay, the organoids from mouse tumors or normal tissue were co-cultured with opT cells. The supernatants from the co-culture were harvested, and secreted IFN-γ was quantified by IFN-γ ELISA kit (Mabtech, 3421–1 H-6). Please see online supplemental methods for further details.

## Results

### Organoid-PBMC co-culture results in generation of organoid-primed cytotoxic T cells

We generated tumor organoids from randomly-selected patients with metastatic pancreatic cancer and classified as classical or basal or mixed subtypes (online supplemental table S1A) were grown in Pancreas Tumor Organoid Media (PTOM) as described previously.^16^ The organoid cultures were comprised of tumor epithelia as determined by H&E staining (online supplemental figure S1A). Autologous PBMCs were cultured in a HTM (see Methods) for 10 days to enrich for lymphocytes relative to monocytes, as the latter cell type may present antigens found in the Matrigel, human serum, and/or other medium components. The HTM media did not include CD28 agonist, which can educate naïve T cells to recognize self or non-specific antigens in vitro.^22^

10.1136/jitc-2021-003213.supp6 Supplementary data

10.1136/jitc-2021-003213.supp7 Supplementary data

**Figure 1:**
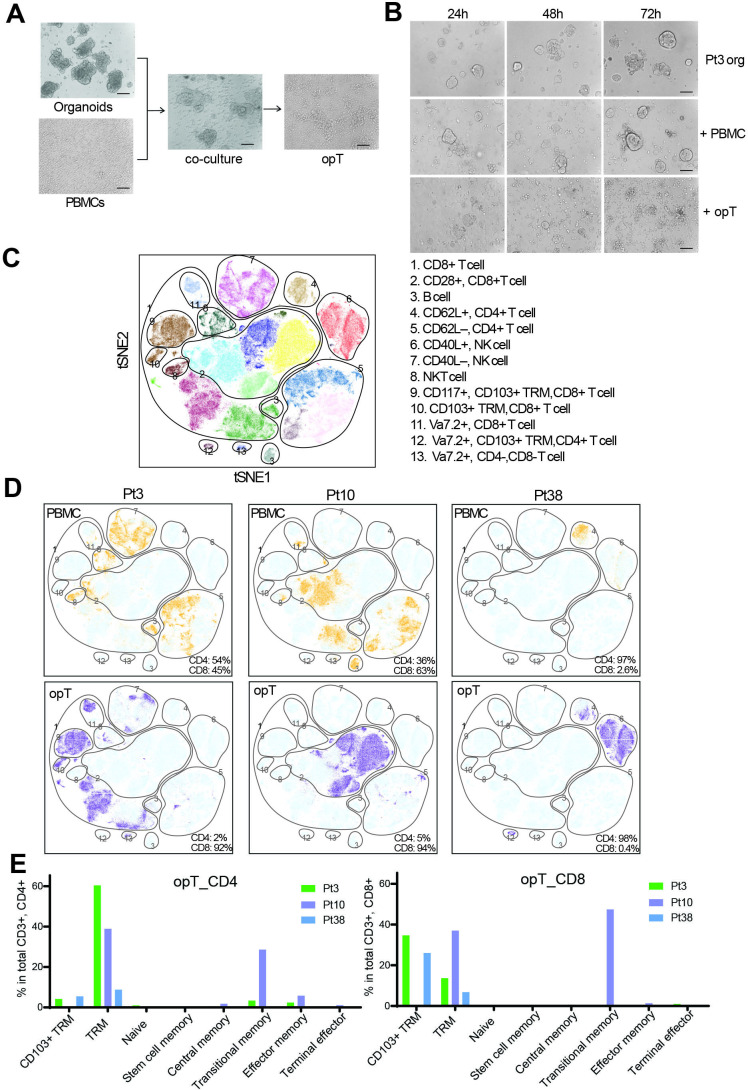
Generation and characterization of tumor-targeting cytotoxic T cells using autologous tumor organoids. (A) Patient tumor organoids co-cultured with autologous PBMC for 14 days for generation of organoid-primed T (opT) cells. Scale bar, 100 µm. (B) Representative phase-contrast images for Pt3 tumor organoids alone or co-culture with autologous PBMC or opT at different time points to demonstrate cell killing. Scale bar, 100 µm. (C) t-SNE plot showing all possible clusters detectable by CyTOF analysis of the six samples. The CyTOF clusters were assembled into 13 phenotypic groups for interpretation. (D) Comparison of PBMC (yellow) and opT (purple) cells for the presence of CyTOF clusters against the background of all possible clusters (light blue). The percentage of CD4 +and CD8+ cells within the CD3 + population is shown in the bottom right. (E) Phenotypes in CD4 + or CD8+ opT cell populations grouped per marker expression. See online supplemental table S2A for the makers used to define the phenotypes. CyTOF, cytometry by time-of-flight; h, hours; NK, natural killer; PBMCs, peripheral blood mononuclear cells; Pt, patient; TRM, tissue-resident memory; t-SNE, t-distributed Stochastic Neighbor Embedding.

10.1136/jitc-2021-003213.supp4 Supplementary data

For co-culture of organoids and PBMC, tumor organoids grown in PTOM for 5–6 days were partially digested from Matrigel (see Methods) to retain their 3D architecture and co-cultured with PBMC-derived lymphocytes in HTM media for 2 weeks (see online supplemental method). Co-culture, but not when organoids cultured alone in HTM, resulted in the complete killing of the tumor organoids and the generation of opT cells (figure 1A). The total number of opT cells generated by co-culture varied between patients studied. The Patient 10 (Pt10) co-culture yielded 3.4 million opT cells, whereas Pt3 co-culture yielded 216 million opT cells (online supplemental table S1B), suggesting patient-specific differences in inducing T cell expansion. When re-challenged with autologous tumor organoids, opT cells killed tumor cells efficiently within 24–48 hours, demonstrating their cytotoxicity (figure 1B and online supplemental figure S1B, C).

The lack of available normal tissue from patients with pancreatic ductal adenocarcinoma (PDAC) prompted us to consider alternative models to investigate the tumor-specificity of opT cells. We co-cultured organoids generated from mammary tumors of the BRCA1/TP53 tumor model with splenocytes expanded from matched tumor-bearing animal. These murine opT cells effectively induced IFN-γ secretion and tumor-cell killing but did not mount a cytotoxic response against normal mammary epithelial organoids generated from syngeneic mice, demonstrating tumor selectivity for opT cell-mediated killing (online supplemental figure S1D, E). opT cells exist in a memory state with patient-specific differences in cell surface markers

To understand the cell types present in opT cells, we assessed the expression levels of 28 cell surface proteins at a single-cell level by mass CyTOF analysis (online supplemental table S1C). CyTOF results from Pt3, Pt10, and Pt38, were analyzed using a t-distributed Stochastic Neighbor Embedding (t-SNE) and an unbiased clustering algorithm to find groups or clusters of cells with similar expression profiles of the 28 markers. The analysis, represented as a t-SNE plot (figure 1C), identified 13 subgroups representing distinct cell types and lineages (figure 1D). PBMCs from all three patients clustered into multiple phenotypic groups, dominated by T cells (CD3+, CD4 + and/or CD8+) with minor populations of B cell and natural killer (NK) cell types (figure 1D). OpT cells from Pt3 and Pt10 were comprised of primarily CD3 + T cells (89%–90%), whereas Pt38 opT had 33.8% CD3 + T cells (figure 1D). Among the CD3 + cells, Pt3 and Pt10 opT cells were CD8 + cells whereas, Pt38 opT cells were primarily CD4+ (figure 1D). Pt19 opT cells were CD3 + made up of 42% CD8 + and 36% CD4 + cells (online supplemental figure S1F). All four patients had stage IV metastatic disease with mutations in KRAS and TP53 at the time of diagnosis suggesting that the patient-specific differences in the opT cell phenotype may be related to differences in other features of the individual patients. Despite Grade 2 disease, Pt38 had the shortest survival time after diagnosis (6 months) (online supplemental table S1A). Although the absence of tumor-targeting CD8 + cells in the circulation may relate to the poor clinical outcome for Pt38, further studies are needed to test this possibility.

OpT cells from all three patients expressed markers of tissue-resident memory (TRM) T cells or CD103 + TRM (figure 1E, online supplemental table S2A). CD103 interacts with E-cadherin, which was expressed on tumor epithelia and is also associated with a T cell activation phenotype in human solid tumors^23^ suggesting that CD103 expression in opT cells may mark both memory and activation states. There was patient-to-patient variation in the type and percentage of cells with a memory phenotype (figure 1E), but T cells with naïve or exhausted phenotypes were in uniformly low abundance. The patient-to-patient differences demonstrate that the culture condition does not normalize the differentiation status of opT cells. Thus, our platform represents a unique opportunity to generate personalized opT cells with a memory phenotype for use in adoptive cell therapy applications in the clinic.

### CD8+ and CD4+ opT cells kill tumor cells in granzyme B or FAS ligand-dependent manner

**Figure 2:**
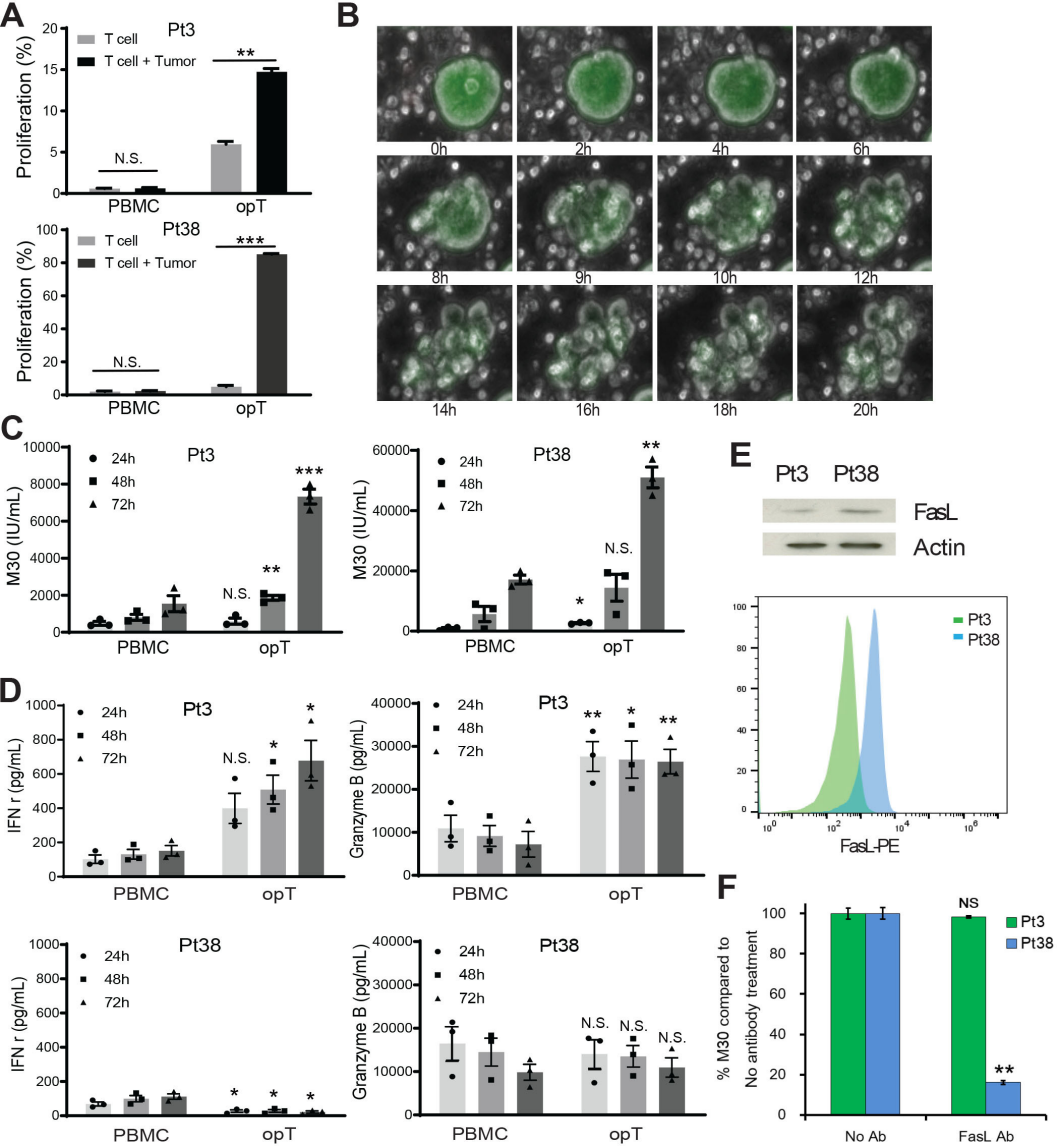
Cytotoxic activity of opT cells. (A) CFSE (carboxyfluorescein succinimidyl ester) labeled PBMC and opT cell proliferation were co-cultured with autologous tumor organoids for 4 days and changes in percentage of CFSE-low T cell population shown. Mean±SEM from three independent experiments shown. N.S., not significant. **, p<0.01. ***, p<0.001. P value calculated using two-tailed, unpaired t-test. (B) Time lapse image of fluorescently labeled organoids (green) incubated with unlabeled opT cells over a period of 20 hours. Scale bar, 100 µm. (C) Changes in levels of epithelial cell-specific caspase-cleaved cytokeratin 18 (CK18) fragments containing the CK18Asp396 (‘M30’) neo-epitope in the media, quantitated by ELISA. Mean±SEM from three independent experiments shown. Each dot represents the mean of three technical replicates from independent experiments. N.S., not significant. *, p<0.05. **, p<0.01. ***, p<0.001. P value calculated using two-tailed, unpaired t-test. (D) Interferon gamma (IFN-γ) and granzyme B produced by PBMC or opT in the presence of autologous tumor organoids at different time points for Pt3 and Pt38. Mean±SEM from three independent experiments shown. Each dot represents the mean of at least two technical replicates from independent experiments. N.S., not significant. *, p<0.05. **, p<0.01. P value calculated using two-tailed, unpaired t-test. (E) FasL expression in opT cells from Pt3 and Pt38 by immunoblot or flow cytometry. (F) Relative M30 production from the supernatants of Pt38 or Pt3 co-cultured in the presence or absence of anti-FasL blocking antibody for 72 hours. NS, not significant. **, p<0.01. P value calculated using two-tailed, unpaired t-test. FasL, Fas-Fas ligand; h, hours; opT, organoid-primed T; PBMCs, peripheral blood mononuclear cells; Pt, patient.

We next assessed how opT cells and PBMCs differed in their ability to respond to autologous tumor cells. PBMC did not show a significant increase in cell division, as determined by flow cytometry analysis of carboxyfluorescein succinimidyl ester (CFSE) labeled cell populations. In contrast, opT cells showed a 15 or 89% increase in the proliferative CFSE-low population (figure 2A), demonstrating that opT cells respond to autologous tumor organoids by entering the cell cycle.

In addition to proliferation, opT cells displayed other features of activation on co-culture with tumor organoids. Live-cell imaging demonstrated that opT cells made contact with organoids within 2 hours and initiated killing by 8 hours (figure 2B). Tumor cell killing was further validated by ELISA for the epithelial-specific, caspase-cleaved, cytokeratin 18 (CK18) neo-epitope CK18Asp396 (‘M30’); this ELISA was utilized as an alternative to other cell killing assays such as chromium release or viable cell counting, as the 3D structure of the organoids precluded the use of these techniques. M30 levels increase threefold to fivefold (figure 2C), indicating a significant increase in the antitumor cytotoxicity of opT cells relative to naïve PBMCs.

**Figure 3:**
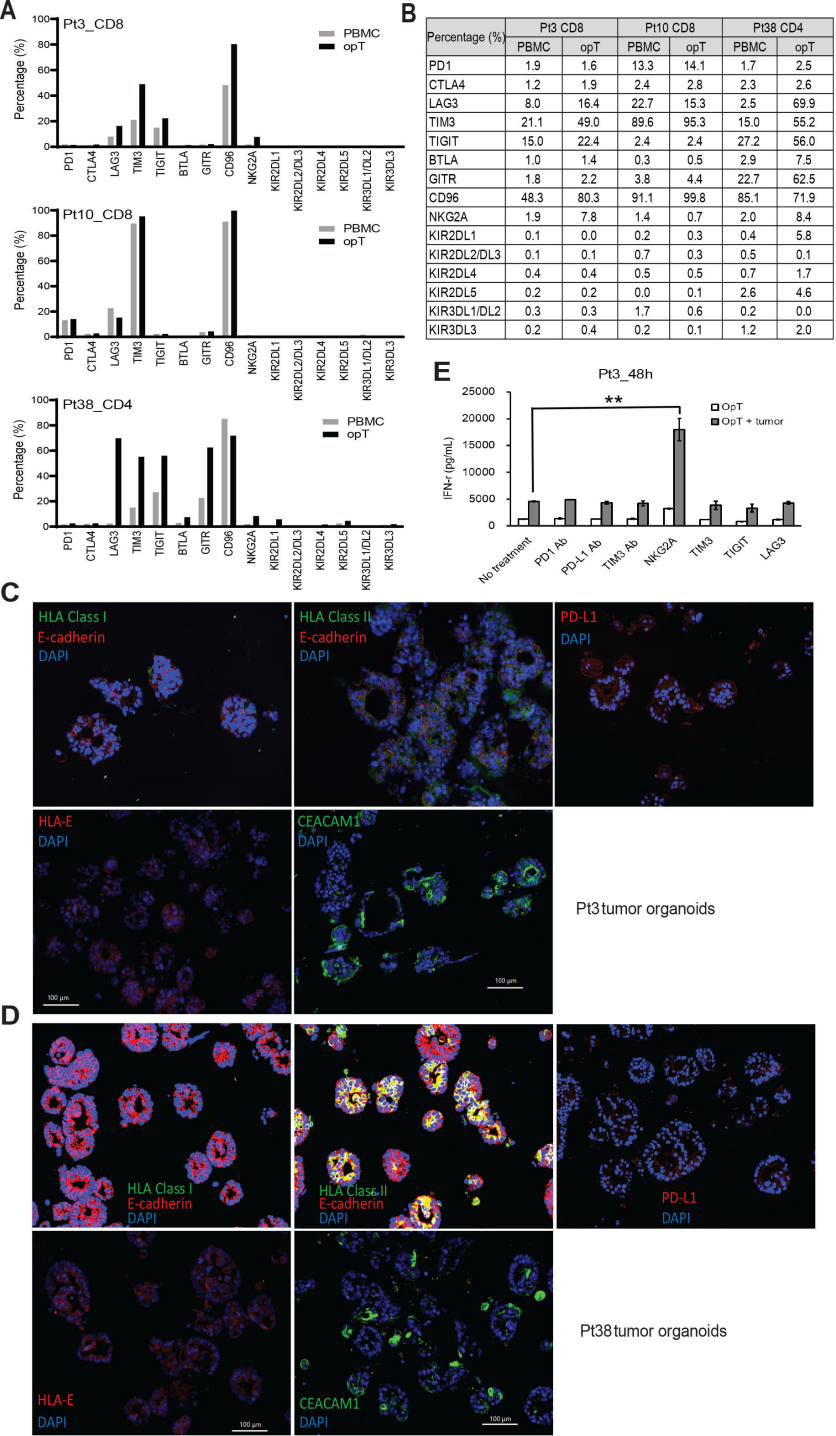
Expression of immunomodulatory proteins and response to checkpoint inhibition in opT cells. (A, B) Expression of checkpoint proteins in opT cells compared with the matched PBMCs by flow cytometry and CyTOF. (C, D) Immuno-staining for MHC-I, MHC-II, PD-L1, HLA-E and CEACAM1 in tumor organoids from Pt3 and Pt38. (E) Changes in interferon-γ secretion by opT cells after 48 hours of pretreatment with anti-PD-1, PD-L1 and TIM3 locking antibodies (10 µg/mL) or NKG2A, TIM3, TIGIT and LAG3 protein (2 µg/mL), in the presence or absence of autologous tumor organoids from Pt3. **, p<0.01 compared with opT plus tumor without antibody treatment. P value calculated using two-tailed, unpaired t-test. CyTOF, cytometry by time-of-flight; opT, organoid-primed T; PBMCs, peripheral blood mononuclear cells; Pt, patient.

The Pt3 opT cells were significantly better than PBMC in secreting IFN-γ and granzyme B when exposed to autologous tumor organoids (figure 2D), consistent with their cytotoxic abilities. Interestingly, the Pt38 opT cells did not differ from PBMC in secretion of granzyme B or IFN-γ (figure 2D), suggesting that Pt38 opT cells kill tumor cells by alternate mechanisms. As shown in figure 1D,~60% of Pt38 opT cells expressed NK cell markers so we tested if the lack of IFN-γ secretion was due to the presence of NK cell. We sorted CD3 + opT cells and investigated their response to organoids. The CD3 + cells opT cells failed to secrete IFN-γ demonstrating that lack of IFN-γ secretion was not due to the presence of NK cells (online supplemental figure S2A). Pt38 CD3 + opT were primarily CD4 + T cells (figure 1D), and cytotoxic CD4 + cells are known to kill tumor cells by FasL-mediated mechanisms.^24 25^ Pt38 opT cells had higher expression levels of FasL than Pt3 (figure 2E), and anti-FasL antibody significantly inhibited killing mediated by Pt38 opT cells with no effect for Pt3 opT cells. These results demonstrate that the CD4 + opT cells from Pt38 kill tumors using the FasL pathway (figure 2F). CD4 + T cells recognize antigens presented by human leukocyte antigens (HLA) class II receptors, consistently, the Pt38 tumor cells express HLA class II proteins (figure 3D), suggesting that CD4 + T cells in Pt38 opT may directly recognize the antigens presented by tumor cells.

10.1136/jitc-2021-003213.supp8 Supplementary data

### opT cells provide insights into patient-specific differences in checkpoint regulation

In addition to TCR engagement with the antigen on tumor cells, T cells are regulated by receptors and ligands that can stimulate or inhibit their function.^26^ These co-stimulatory or co-inhibitory (also known as checkpoint inhibitors) are required for maintaining immune homeostasis in normal tissues.^26^ In cancer tissues, an increase in expression of checkpoint inhibitors is frequently associated with immune evasion, and blockade of these pathways is an established strategy for cancer immunotherapy.^27^ We monitored expression levels of 17 immune checkpoint-inhibitors, including the frequently studied PD-1, CTLA-4, TIM-3, TIGIT, CD96, LAG-3, BTLA, GITR, NKG2A, and KIRs (KIR2DL1-5 and KIR3DL1-3)^27^ in both PBMC and opT cell populations. Pt3 opT cells showed increased expression of LAG3, TIM3, TGIT, CD96, and NKG2A; Pt10 opT had limited changes in expression of all checkpoint proteins tested, and Pt38 opT cells showed an increased expression of LAG3, TIM3, TIGIT, BTLA, GITR, NKG2A, and KIR2DL1 (figure 3A and B) demonstrating intr-patient variations in the expression of checkpoint inhibitors. We next monitored changes in the expression levels of ligands for the immunomodulatory receptors on tumor cells. HLA class II (ligand for LAG3), PD-L1 (ligand for PD-1), HLA-E (ligand for NKG2A), and CEACAM1 (ligand for TIM3) were all expressed on tumor organoids (figure 3C, and D online supplemental figure S2B), suggesting that the opT cells were likely to be regulated by these checkpoint proteins. To determine the functional significance of checkpoint protein expression, we used inhibitory antibodies or soluble proteins to block PD-1, PD-L1, TIM3, TIGIT, LAG3, and NKG2A receptors. NKG2A blockade showed the most potent increase in the IFN-γ production 24 and 48 hours after stimulation (figure 3E, online supplemental figure S2C) compared with blocking other ligands. Although NKG2A is primarily expressed on NK cells, it is also detected on CD8 + T cells within the tumor environment.^28^ Consistently, sorted populations of CD3 + opT cells retained the ability to respond to NKG2A blockade (online supplemental figure S2D, E), demonstrating that NKG2A regulates cytotoxic T cell activity of Pt3 opT cells. Interestingly, expression of an NKG2A ligand, HLA-E expression, is associated with poor prognosis in pancreatic cancer.^29^ These results demonstrate the ability to use the co-culture platform to identify checkpoint inhibition strategies for effective immunotherapy.

### opT cells undergo dramatic clonal expansion

**Figure 4:**
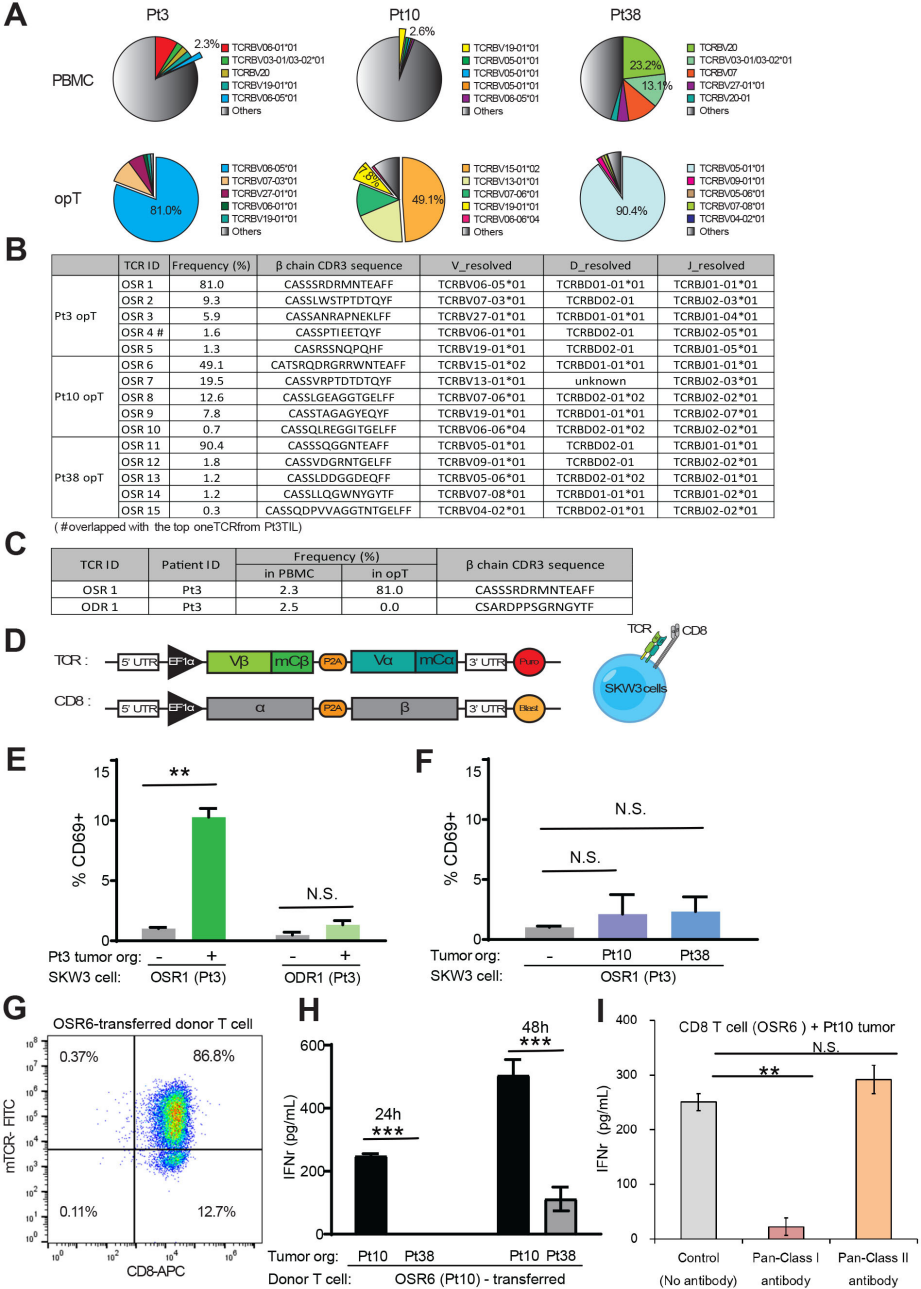
Clonal expansion in opT cells and identification of TCR. (A) Pie-charts showing representation of a given TCR beta-chain as a percentage of the all the independent TCRs detected. (B) Frequencies and CDR3 beta-chain sequences for the top five organoid-selected TCR (OSR) from each patient. (C) TCR enriched in Pt3 opT and organoid-depleted TCR (ODR1) used for generation of recombinant TCR. (D) A schematic of chimeric TCR that comprised of human Vα and Vβ chains and mouse constant α and β chains and co-expressed with human CD8 gene in SKW-3 cells. (E) Expression of T cell activation marker (CD69) in TCR-expressing SKW-3 cells exposed to autologous (Pt3) tumor organoids. N.S., not significant. **, p<0.01. P value calculated using two-tailed, unpaired t-test. (F) Expression of CD69 in OSR1 expressing SKW-3 cells exposed to allogenic (Pt10 and Pt38) tumor organoids. N.S., not significant. (G) CD8 + T cells from a donor PBMC were transduced with the chimeric TCR (OSR6) and flow sorted by mouse-TCR chimera (mTCR-FITC) expression. (H) OSR6 expressing CD8 + T cells were exposed to autologous (Pt10) or allogenic (Pt38) organoids and media analyzed for IFN-γ by ELISA. ***, p<0.001. P value calculated using two-tailed, unpaired t-test. (I) OSR6 expressing CD8 + T cells were exposed to autologous (Pt10) organoids with either Pan-MHC Class I antibody (W6/32) or Pan-MHC Class II antibody (Tu39) and media analyzed for IFN-γ by ELISA. N.S., not significant. **, p<0.01. P value calculated using two-tailed, unpaired t-test. IFN, interferon; opT, organoid-primed T; PBMCs, peripheral blood mononuclear cells; Pt, patient; TCR, T cell receptor.

To investigate if opT cells are clonally expanded populations of tumor-targeting T cells, we sequenced TCR for matched PBMC and opT cells from Pt3, Pt10, and Pt38. Although both RNA and DNA can be used to identify TCRs, we chose to use DNA as a template for sequencing reactions because copies of RNA expression can vary significantly between cells, thus creating challenges for the precise determination of clonal frequencies in a given cell population. Among the 150,000 or more TCR β-chains sequenced, no TCR was represented more than 3.0% of PBMC from either Pt3 or Pt10, demonstrating the polyclonal nature of the population (figure 4A). However, opT cells contrasted from PBMCs in that they were strongly oligoclonal. Pt3 opT cells were dominated by one TCR (referred to as Organoid Selected T cell Receptor-1, OSR-1), which represented 81% of the opT cell population, while four other clones contributed to an additional 18% of this population (figure 4A, B). OSR1 underwent more than a 35-fold clonal expansion (2.3% in PBMC to 81% in opT cells) after the co-culture with autologous tumors. In Pt38, the T cell clone OSR11 was undetectable in PBMC but was enriched to 90.4% in opT cells, demonstrating a more than 270,000-fold clonal expansion after the initial co-culture priming. Thus, in each of the three opT cell populations, five TCRs made up 89.8% to 99.0% of the diversity in the >150,000 TCRs analyzed, demonstrating an unexpected and powerful clonal expansion. Furthermore, all the 15 highly expanded TCRs were distinct, demonstrating antigen diversity among these patients. TCRs identified in the opT population of one patient with cancer were not present in the PBMC of the other patients with PDAC suggesting that there may be significant patient-specificity (online supplemental table S2C). It is also possible that TCRs are shared between patients, which may require analysis of a larger cohort of opT cells.

### TCRs expressed in opT cells are shared with TILs but show limited prevalence in healthy individuals

To understand how the TCRs selected in our co-culture platform compared with the T cells naturally enriched in the tumor bed in vivo, we compared the TCRs identified in Pt3 TILs expanded in vitro with Pt3 opT cells. There was a 50% overlap among the top 10 TCRs between TILs and opT cells (online supplemental table S2B), including OSR4 (figure 4B), showing that there are both similarities and differences in the clonal selection that occurs in the tumor bed versus the co-culture platform. Since the co-culture lacks inhibitory factors contributed by the microenvironment, it likely creates an opportunity for unrestricted T cell-tumor cell interactions, contributing to the observed difference between TCRs from opT cells and TILs.

We compared the β chain of the top 5 TCRs from the opT cells and TlLs with a TCR database from 120 healthy individuals to determine if the TCRs expressed by opT cells were present in healthy individuals. Among the 20 TCRs we selected from opT cells, 1 TCR (OSR11, from Pt38 opT cells) was present in 25 healthy individuals with the highest frequency of 1.1×10^–5^, 4 other TCRs were present in less than 10 individuals, and 15 were not observed in any of the 120 healthy individuals (online supplemental table S2D). These analysis demonstrate that the TCRs identified in opT cells are likely to be tumor-selective and not prevalent in healthy individuals.

### TCRs identified in opT cells are sufficient to confer tumor cell-targeting ability to heterologous T cells

Next, we investigated if the TCRs expressed on opT cells can confer tumor recognition when expressed in heterologous T cells. We determined the sequence of both the alpha and beta chains of the TCRs in opT cells and selected the top five TCRs to generate a chimeric TCR (figure 4B). TCR sequences that were detectable in PBMC but lost during the opT selection process (Organoid Depleted T cell receptors (ODR)) were selected to serve as negative controls (figure 4C). Either ODR or OSR chimeric receptors were expressed in SKW-3 cells, a T cell line that lacks endogenous TCR,^30^ and these SKW-3 cells were then exposed to organoids to monitor changes in the expression of the activation marker CD69. The SKW-3 cells expressing the positively-selected TCRs induced a significant increase in the expression of CD69 on co-culture with autologous (figure 4E) but not allogeneic tumor organoids (figure 4F), highlighting the presence of patient-specific antigens that are recognized by the TCRs selected in opT cell populations. Unlike the OSR11 beta chain (which constituted 90.4% of Pt38 opT TCRs), we observed two alpha chains at 46% each, suggesting that the OSR11 beta chain may exist as two different TCR pairs. SKW-3 cells expressing the OSR11-2, but not the OSR11-1 chimera, increased the expression of CD69 on co-culture with Pt38 organoids, demonstrating that only one of the two α/β pairs was functional in recognizing antigens on Pt38 tumor cells (online supplemental figure S3A). Thus, we demonstrated that the TCRs expressed on empirically expanded CD8+ (Pt3) or CD4+ (Pt 38) opT cells were sufficient to confer tumor-recognition ability in a patient-specific manner.

10.1136/jitc-2021-003213.supp9 Supplementary data

Next, we investigated if chimeric OSRs can be used to engineer primary T cells and confer cytotoxic ability because the use of engineered T cells expressing cancer-targeting TCRs or chimeric antigen receptors for immunotherapy has gained significant attention.^31^ PBMC-derived CD8 + T cells from a healthy donor were engineered to express a chimeric TCR generated with OSR6, the dominant TCR identified in Pt10 opT cells (figure 4B). We achieved more than 85.0% transduction efficiency as determined by TCR flow analysis (figure 4G). Incubation of the engineered T cells with autologous (Pt10) organoids induced IFN-γ secretion within 24 hours, which increased by 48 hours. Incubation with allogenic (Pt38) organoids did not induce detectable IFN-γ secretion during the first 24 hours (figure 4H), but an detectable increase in IFN-γ secretion was detected by 48 hours (figure 4H). The delayed response to allogenic organoids was likely due to an HLA-mismatch mediated response. An MHC class I blocking antibody inhibited IFN-γ secretion, demonstrating that the tumor recognition by TCRs is MHC-dependent (figure 4I). These observations directly demonstrate the ability of the TCR identified in opT cells was sufficient to confer the tumor-targeting ability to engineered-primary human T cells.

## Discussion

Here we report the development and characterization of a platform to identify and expand tumor-targeting cytotoxic T cells from the circulation of patients with pancreatic cancer by co-culturing PBMCs with autologous tumor-derived organoids. These organoid-primed T cells underwent clonal expansion, expressed TRM phenotype markers, and were highly effective in killing autologous tumor organoids. There is a significant need to develop new immune-oncology approaches to treat pancreatic cancer because pancreatic cancer is non-responsiveness to immune checkpoint inhibitors.^32 33^ Since the TRM phenotype is associated with the T cells that have infiltrated into tissues and is considered essential for immunity in solid tumors,^34 35^ our organoid-PBMC co-culture is likely an effective platform for identifying tumor-targeting T cells for cell therapy. Further experiments will be needed to assess the effectiveness of opT cells for adoptive cell therapy against pancreatic cancer.

The opT cell platform can provide new insights into the mechanisms regulating pancreatic tumor T cells interactions. For example, the opT cells from Pt38 were CD4 + cytotoxic T cells that killed tumor cells in a FasL dependent manner. Although there is precedent for cytotoxic CD4 + T cells in PDAC,^36^ CD8 + T cells are the most commonly studied cell type, but our observations demonstrate that the opT platform is agnostic to the type of cytotoxic T cells it can enrich for and hence can effectively provide new insights into cytotoxic T cells against PDAC.

It is likely that the ineffectiveness of checkpoint inhibition therapy in PDAC^32 33^ is due to an incomplete understanding of the checkpoint regulators in PDAC. Our results suggest a role for the NKG2A-HLA-E axis in PDAC. Consistent with our observations, a recent study identified an association between HLA-E expression and poor survival.^29^ Further studies would be needed to determine if the HLA-E-NKG2A checkpoint axis has therapeutic utility for patients with PDAC.

Identifying T cell receptors that are tumor-selective with limited or no cross-reactivity to normal cells will be a significant advance towards identifying new immune therapy approaches for solid tumors. Here, we demonstrate our ability to achieve a high degree of clonal expansion of cytotoxic T cells from PBMC and identify tumor-targeting T cell receptors. The identified TCRs are sufficient to elicit patient-specific tumor recognition when expressed in allogeneic T cells. Taken together, we demonstrate the utility of our platform for the empirical identification of TCRs that can be used in TCR-therapy applications for patients with pancreatic cancer.

## Data Availability

Data are available in a public, open access repository. All data relevant to the study are included in the article or uploaded as supplemental information. All TCR data will be made available upon acceptance of the manuscript.
