# Correction: Empirical identification and validation of tumor-targeting T cell receptors from circulation using autologous pancreatic tumor organoids

**DOI:** 10.1136/jitc-2021-003213corr1

**Published:** 2023-05-15

Meng Q, Xie S, Gray GK, *et al*. Empirical identification and validation of tumor-targeting T cell receptors from circulation using autologous pancreatic tumor organoids. *J ImmunoTher Cancer* 2021; 9:e003213. doi: 10.1136/jitc-2021-003213.

This article has been corrected since it was first published. Omar Gandarilla was mistakenly left off the author list and has now been added.

