# Supplementary material for: Empirical identification and validation of tumor-targeting T cell receptors from circulation using autologous pancreatic tumor organoids
Source: J Immunother Cancer. 2021 Nov 16;9(11):e003213. doi: 10.1136/jitc-2021-003213 (PMC8601084; doi:10.1136/jitc-2021-003213)
Supplement: Supplementary data [file jitc-2021-003213supp001.pdf]

# Supplement methods

## Generation of human pancreatic tumor organoid-primed T cells (opT)

**T cell preparation** Peripheral blood mononuclear cells (PBMCs) were isolated from the patients' peripheral blood by Ficoll-paque density, and then cultured for 10 days in human T cell medium (HTM): Serum-free medium (CellGernix, 20801-0100), 10% human AB serum (Innovative Research, IPLA-SerAB-13458), human IL-2 (1000IU/ml, Prospec, cyt-209), human IL-15 (10ng/ml, Prospec, cyt-230), human IL-21 (10ng/ml, Prospec, cyt-408), 1% penicillin-strepomycin (Gibco, 15140-122), 1% amphotericin B solution (Sigma, A2942) and ciprofloxacin (Fisher Scientific, 50255729).

**Tumor organoids preparation** Organoids were growing in 12 well plate for 5-6 days. The organoids were partially digested from Matrigel by Collagenase/Dispase (Sigma, 11097113001) with 1.5 hours at 37°C and retain their 3D architecture. These organoids were washed with human T cell medium to remove Matrigel and used for co-culture. To determine cell count, part of the organoids were digested to single cells by TrypLE™ Express Enzyme (Thermo Fisher, 12605028) and counted.

**Tumor organoid-T cell Co-culture** After 10 days of culture, 100,000 PBMCs were co-cultured with 100,000 autologous tumor cells (with organoid 3D structure) per well in a 96 well flat bottom plate (Falcon, 353072) for a total of 3 wells in human T cell medium. At day 7, the cells were transferred to a new 24 well plate (Falcon, 353047) and the PBMCs were stimulated again with same autologous organoids at a ratio of 1:1 for another 7 days. PBMCs without tumor stimulation were set up as a control.

## Generation of tumor-infiltrating lymphocytes (TIL)

TIL generation were performed as previously described [20]. Tumor were minced into 1-2 $mm^2$ fragments and cultured with human T cell medium including cytokines to expand TILs. After 10 days of culture, TILs were stimulated with anti-CD3 antibody (OKT3) with irradiated, allogeneic PBMC as feeder cells. The feeder cells from mixed 5 donors were irradiated by 55 Gy and used at a ratio of 1:10 (TIL : feeder cell).

**Live imaging**

After the organoids were passaged for 7 days, the Matrigel in the culture was digested by collagenase/dispase while keeping the 3D structure of organoids intact. The organoids we labeled with CellTracker™ Green CMFDA Dye (Thermo Fisher, C7025) and 0.1 $x10^6$ cells in 500 L of PTOM media were transferred to a well of 24 well plate. Three hours later, one million opT cells in 500 L human T cell medium were added into the same well. Images were taken every 10 minutes starting at 2.0 hour co-culture for a total of 20 hours by fluorescence microscope (KEYENCE, BZ-X810) at 37 degree, 5% CO2.

**3D killing assay and IFN-γ and Granzyme B production in 3D co-culture**

Organoid cultures were digested into single cells, and plated onto Matrigel coated well of 96-well flat bottom plate at the rate of 10,000 cells in 100 l of PTOM media per well. After 4 days of growth to allow formation of organoid structures, autologous PBMC or opT cells resuspended in 100 l of fresh T cell medium (IL-2, 50 IU/ml, Prospec) were added to tumor organoids at a cell ratio of 1:1, with final media containing 50:50 ratio of PTOM and HTM. To get the correct ratio after 4 days, extra wells of tumor organoids were digested into single cells and counted before adding the T cells. Cells were incubated at 37°C for 24h, 48h and 72h, respectively, after which supernatants were collected and stored at −80°C until further analysis. Target cell apoptosis was measured using enzyme-linked immunosorbent assay (ELISA) for the epithelial cell–specific

caspase-cleaved cytokeratin 18 (CK18) fragments containing the CK18Asp396 ('M30') neo-epitope (PEVIVA), according to the manufacturer's instructions, M30 Apoptosense® CK18 Kit (DiaPharma, P10011). The supernatants were also used to measure IFN-γ and Granzyme B by ELISA as per the manufacturer's instructions: IFN-γ ELISA kit (Mabtech, 3420-1H-20) and Granzyme B ELISA kit (Mabtech, 3485-1H-20).

**FasL expression by western blot**

Cells were lysed in cell lysis buffer supplemented with protease inhibitor cocktail (Roche#04693159001) and phosphatase inhibitor (Roche#04906837001). Twenty micrograms of protein was separated in 4–15% Mini-PROTEAN TGX Precast Protein Gels by SDS PAGE electrophoresis and transferred to onto PVDF membranes (IPVH00010, EMD Millipore). Membranes were blocked in 1.0 % BSA (Sigma) dissolved in TBS-T for 1.0 hour and incubated overnight at 4°C with primary antibody: mouse anti-human monoclonal Fas-L (Biolegend#306409) or mouse anti-human monoclonal β-actin (Sigma#A5441). Membranes were washed three times with TBS-T and incubated with secondary antibody for 1.0 hour at room temperature: horseradish peroxidase (HRP)-conjugated anti-mouse IgG (GE healthcare) and imaged.

**FasL expression by Flow Cytometry**

Pt3 opT and Pt38 opT cells were stained with Live/Dead marker Zombie Violet (Biolegend, 77477), CD3 APC (Biolegend, 300312) and FasL PE (Biolegend, 306406). CD3+, alive T cells were gated and FasL staining positive cells are shown in the histogram. Data was analyzed with FlowJo v10.

**Phenotype and memory marker test by CyTOF**

Twenty eight mass-labeled cell surface antibodies were purchased from Lederer's laboratory (Brigham and Women's Hospital, Harvard Medical School, Boston, MA) and their detailed information were included in Table S1. Cell staining was performed as Maxpar Cell Surface Staining with Fresh Fix (Fluidigm). Briefly, opT or PBMC cells were first stained with Cell-ID Cisplatin, and incubated with Fc receptor blocking solution; Cells were then stained with the surface antibodies, incubated for 15 minutes at room temperature, and fixed with fresh 1.6% formaldehyde; the cells were stained with Cell-ID Intercalator-Ir, and EQ beads were added before acquisition. The samples were tested by Helios with WB Injector at Dana-Farber mass cytometry core. After performing quality control to remove dead cells, debris, and other low-quality events according to the standards of the field [36], the remaining high-quality events were used to generate t-SNEs using Scanpy [37], and clustering based on the expression of all antibodies tested was performed using the Leiden algorithm [38] .

**TCRα, β and δ chain sequencing**

Three million opT or PBMC cells (PBMCs were cultured in same medium, same time as opT but without tumor stimulation) were used for genomic DNA extraction by QIAamp DNA Blood Mini Kit (Qiagen, 51104). TCR sequencing survey (ImmunoSEQ) was performed by Adaptive Biotechnologies on genomic DNA. Only productive TCR rearrangements were used in the calculations of TCR clonotype frequencies. TCRα-TCRβ chains were sequenced separately using the same DNA sample: the TCRβ were sequenced by immunoSEQ hsTCRB v4 and TCRα by immunoSEQ hsTCRA/D. Total productive TCR reads per sample varied between 157,295 to 283,448. The data were analyzed with Immanalyses 3.0 (ImmunoSEQ).

**TCR construction, transduction and purification**

*TCR Construction*

By the TCR production ratio to identify the matched TCRA-TCRB chain.

Full-length TCR-encoding sequences were constructed by fusing the CDR3 region of a putative reactive TCR (provided by ImmunoSEQ sequencing analysis) with the missing conserved V- and J-region-encoding sequences obtained from the IMGT online database (http://www.imgt.org). The sequence encoding the resulting human V-(D)-J region was fused to the modified mouse TRAC or TRBC region. Sequences of mouse constant region for both α and β chains were fused to Vα and Vβ sequences, respectively. The self-cleaving peptide P2A was added between VβmCβ and VαmCα as a linker. The integrated amino acid sequence was converted to DNA sequence using the Codon Optimization Tool (https://www.idtdna.com/CodonOpt). Recombinant TCR sequence was then flanked with attB1 and attB2 sequences at both ends. The full length was synthesized and final recombinant TCR expression vector was assembled by Gateway cloning and subcloned into pHAGE EF1α-Puro.

*TCR transduction*

Packing virus: One 6 well plate of HEK293T cells was transfected with TCR encoding pHAGE plasmid(s) (0.5 g/well) and the envelope-encoding plasmid Lenti-Pack Mix (4.6 l/well, Sigma) using FuGene 6 (Promega, E2691). Retroviral supernatants were collected at 48 hours after transfection.

Infection: one million SKW3 cells were infected with 2.0 ml virus supernatants, polybrene (final 8 g/ml) and 8.0 ml RPMI with 10%FBS in a 15 ml tube. Centrifuge at 800g for 60 minutes. After the centrifugation, 5.0 ml supernatant was replaced with fresh RPMI+10%FBS.

*TCR purification*

Three days after infection, puromycin (1 g/ml,) was added for two-four days and TCR positive cells were then sorted by FACS using mTCR antibody (Biolegend, 109212).

For human CD8 αβ transduction and purification, cells were selected by blasticidin (final 10 g/ml) after infection.

**SKW3 activation**

Matrigel was digested by collagenase/dispase while keeping the organoids in 3D structure. In a well of a flat bottom 96 well plate, 0.5 million organoids were mixed with 0.1 million SKW3 cells in 200 l of RPMI medium with 10% FBS. Each condition was tested in triplicate. After co-culture for 16 hours, the cells stained with CD3 PE (Biolegend, 300308), CD8 BV510 (Biolegend, 344732), mTCR APC (biolegend, 109212) and CD69 BV605 (Biolegend, 310938) antibodies and analyzed by Flow cytometry. Data were analyzed by flowjo v10.

**TCR transfer to allogenic CD8+ T cells and tumor recognition**

CD8+ T cell were sorted by flow cytometry from healthy donor PBMC and used for transfer with the chimeric TCR vector using a 4D Nucleofector (Lonza Biosciences). After TCR transfer, the T cells were sorted using mouse TCR antibody and beads. The sorted cells were confirmed by CD3 PE (Biolegend, 300308), CD8 APC (Biolegend, 344722) and mTCR (Biolegend, 109206) antibody. To test if the engineered-T cells recognize tumor organoids, 0.1 million organoids per well were co-cultured with 0.1 million engineered-T cells in human T cell medium in a 96 well plate with flat bottom with total 250 l per well After 24h and 48h co-culture, the supernatants were collected and tested for IFN-γ secretion by ELISA (Mabtech, 3420-1H-20). For MHC class I and MHC class II blocking experiment, purified antibodies without azide (final 10 g/ml) were used: Pan-MHC Class I antibody (clone W6/32, Biolegend, 311428), Pan-MHC Class II antibody (clone Tu39, BD Pharmingen, 555556).

**Immune checkpoint related protein expression tested by CytoF and flow cytometry**

Seventeen immune-modulatory proteins have been tested in this study for PBMCs and opT. PD-1 and CTLA-4 were tested by CyTOF (antibody details in Table S1). The rest 15 proteins were stained with 13 antibodies (antibody details in Table S3) and tested by flow cytometry. Based on the limitation of antibody colors, the 13 antibodies were separated into 5 groups and each group was stained with CD3, CD4, CD8 and specific immune regulation markers including Group 1: CD3 PE, CD4 APC, CD8 FITC, NKG2A BV711, LAG3 BV605 and KIR2DL2/DL3 Percp; Group 2: CD3 BV605, CD4 BV650, CD8 FITC, TIM3 APC, KIR2DL1 PE and GITR percp/cy5.5; Group 3: CD3 BV605, CD4 BV650, CD8 Percp, KIR2DL5 PE, KIR3DL3 AF647, LIR2DL4 FITC; Group 4: CD3 BV605, CD4 BV650, CD8 Percp, TIGIT APC, BTLA PE, KIR3DL1/DL2 FITC; Group 5: CD3 BV605,CD4 BV650,CD8 Percp and CD96 PE. Data was analyzed with FlowJo v10.

**Analyzing effect of immune checkpoint protein blocking**

Organoids in 3D structure were seeded at the rate of 0.1 million per well in a 96 well plate with flat bottom. The organoids were incubated with indicated antibody or protein for 30 minutes, and then 0.1 million opT cells were added to the same well. Organoids alone and T cells alone were used as controls. Human T cell medium were supplemented with 10 g/ml antibody or 2 g/ml soluble protein: PD1 (Selleckchem, A2002), PD-L1 (Biolegend, 329716), TIM3 (Biolegend, 345038); recombinant protein NKG2A (Prospec, PRO-2440), TIM3 (Prospec, HAV-221), TIGIT (Prospec, PRO-2498) and LAG3 (R&D, 2319-L3-050). The supernatants were harvested at 24 hours and 48 hours, respectively and tested by IFN-γ ELISA kit (Mabtech, 3420-1H-20).

**T cell sorting from opT**

T cells were sorted with human CD3 positive selection kit (Stemcell Technologies, 17851) and cultured in human T cell medium for 14 days to avoid T cell activation. The purity of T cells was

measured by flow cytometry with Live/Dead marker Zombie Violet (Biolegend, 77477), CD3 APC (Biolegend, 300312) and CD56 PerCP/Cy5.5 (Biolegend, 362505). Live cells were first gated and then checked the CD3 and CD56 expression. Data was analyzed with FlowJo software.

**Tumor-induced T cell proliferation by CFSE, flow cytometry**

PBMC and opT cells were first labeled by CellTrace™ CFSE (Thermo Fisher, C34554) and organoids were prepared in 3D structure without Matrigel. 0.1 million organoids per well were co-cultured with 0.1 million T cells in human T cell medium into a 96 well plate with flat bottom. Each condition was analyzed in triplicate wells. After 4 days of co-culture, cells from three wells were pooled together and stained with CD3 PE (Biolegend, 300308), CD4 BV650 (Biolegend, 317435), CD8 APC (Biolegend, 344722) antibodies and tested by flow cytometry. Data was analyzed with FlowJov10.

**Immunohistochemical staining**

To generate organoid tissue sections, they were grown in chamber slides and fixed in 4% PFA for 2 hours followed by incubation with Hematoxylin solution for 10 minutes and washed twice with water. The organoids were scraped and sandwiched between two layers of Histogel (Sigma) using a cryomold and transferred to a tissue cassette followed by fixation in 10% formalin. Primary antibodies: HLA Class I ABC (Abcam, ab70328); HLA Class II DR, DP, DQ (Abcam, ab7856); PDL1 (Abcam, ab239749); CEACAM1 (Abcam, ab108390); HLA-E (Abcam, ab2216); E-cadherin (Cell signaling, 3195S). Secondary antibodies: Goat anti-Mouse IgG AF488 (Invitrogen, A11029); Goat anti-Rabbit IgG AF568 (Invitrogen, A11036); Goat anti-Rabbit IgG AF488 (Invitrogen, A11034); Goat anti-Mouse IgG AF568 (Invitrogen, A11031).

**Generation of mouse organoids**

Triple-negative breast cancer (TNBC) mouse models harboring breast specific Trp53 deletion in combination with Brca1 deletion (K14cre, Trp53$^{flox/flox}$, Brca1$^{flox/flox}$) were established and obtained from Prof. Alex Toker's laboratory (Beth Israel Deaconess Medical Center, Boston, MA)

[21]. Mouse mammary tumors or normal mammary glands were minced with No.22 blades into 1-2 mm fragments then digested with 1mg/ml collagenase/dispase (Roche) for 30 minutes. The digestion was stopped by adding equal volume of 1% BSA in DMEM, then centrifuged at 1500rpm x 5min. Pellets were further digested with Accutase for 30 minutes then collected by centrifugation at 1500 rpm x 5 minutes. Pellets were then resuspended in organoid growth medium containing Y-27632, 5% matrigel, and growth factors. The suspension was seeded onto 12-well plates pre-coated with Matrigel. Culture media was replaced every four days.

**Generation of mouse opT cells**

Mouse T cells from spleen were isolated by EasySep™ Mouse T Cell Isolation Kit (Stemcell Technologies, 19851), the cells were cultured with mouse complete T cell medium: Serum-free medium (CellGernix, 20801-0100), 10% FBS (Glico, 16140071), mouse IL-2 (1000 IU/ml) (Peprotech, 400-02), mouse IL-15 (10ng/ml, Peprotech, 400-24), mouse IL-21 (10ng/ml, Prospec, CYT-033). After 10 days of culture, the T cells were stimulated with autologous tumor organoids twice. Organoids without Matrigel were used for co-culture, keeping the 3D structure. The T cell and tumor cell ratios range from 1:1 to 10:1. After 14 days of culture, the stimulated and expanded organoid primed T cells (opT) were generated.

**Mouse opT killing assayand IFN-γ detection**

Organoids from tumor or normal tissue were cultured in organoids medium for 4 days after passaging, and then co-cultured with opT cells in mouse T cell medium. The final co-culture medium is composed by half mouse organoids medium and half mouse T cell medium. T cell and

organoids ratio is 1:1. Images were taken by microscope (Leica) every 24 hours. The supernatants from co-culture were harvested at days 2 and 4 and secreted IFN-γ was quantitated by ELISA as per the manufacturer's instructions: IFN-γ ELISA kit (Mabtech, 3421-1H-6).

**Supplementary references**

36. Olsen, L.R., M.D. Leipold, C.B. Pedersen, and H.T. Maecker, The anatomy of single cell mass cytometry data. Cytometry A, 2019. 95(2): p. 156-172

37. Wolf, F.A., P. Angerer, and F.J. Theis, SCANPY: large-scale single-cell gene expression data analysis. Genome Biol, 2018. 19(1): p. 15.PMC5802054

38. Traag, V.A., L. Waltman, and N.J. van Eck, From Louvain to Leiden: guaranteeing well-connected communities. Sci Rep, 2019. 9(1): p. 5233.PMC6435756
