# Supplementary material for: Empirical identification and validation of tumor-targeting T cell receptors from circulation using autologous pancreatic tumor organoids
Source: J Immunother Cancer. 2021 Nov 16;9(11):e003213. doi: 10.1136/jitc-2021-003213 (PMC8601084; doi:10.1136/jitc-2021-003213)
Supplement: Supplementary data [file jitc-2021-003213supp002.pdf]

Supplemental figure and table legends

Figure S1 OpT cells recognize tumor but not normal cell. (**A**) Hematoxylin-eosin (H&E) staining of tumor organoids from patient 3, 10, 19 and 38. Scale bar, 100μm. (B, C) Representative images showing that pt19 opT (B) and pt38 opT cells (C) kill autologous tumor organoids more efficiently than PBMC and in a time-dependent manner. Scale bar, 100μm. (D) Representative images for mouse mammary tumor organoids or normal mammary tissue alone or co-cultured with autologous opT cells at different time points (Day 2 and 4) under light microscopy. Scale bar, 100μm. (E) Bar graphs showing the IFN-γ secretion by mouse opT cells after co-cultured with autologous mammary tumors or normal cells for 2 days. (F) Cell Phenotype for PBMC and opT cells from pt19 by flow cytometry.

Figure S2 (**A**) Changes in IFNγ secretion by sorted T cells from pt38 opT after 24h in the presence or absence of autologous tumor organoids from pt38. (**B**) Immuno-staining for MHC-I, MHC-II, PD-L1, HLA-E and CEACAM1 in tumor organoids from pt10. (**C**) Changes in IFNγ secretion by opT cells after 24h of pretreatment with anti-PD1, PDL1 and TIM3 blocking antibodies or NKG2A, TIM3, TIGIT and LAG3 protein, in the presence or absence of autologous tumor organoids from pt3. *, $p<0.05$. p-value calculated using two-tailed, unpaired t-test. (**D**) Sorted populations of CD3+ opT cells from pt3. (**E**) IFNγ secretion by pt3 sorted CD3+ opT cells after 24h of pretreatment with recombinant protein NKG2A (final 2 μg/ml), in the presence or absence of autologous tumor organoids from pt3. N.S., not significant. **, $p<0.01$. p-value calculated using two-tailed, unpaired t-test.

Figure S3 Expression of T cell activation marker (CD69) in TCR-expressing SKW-3 cells exposed to autologous (pt38) tumor organoids. Lower table, α chains details for OSR11-1 and OSR11-2 which shares the same β chain as is shown for OSR11 (Fig. 4B).

Meng Q, *et al. J Immunother Cancer* 2023; 9:e003213. doi: 10.1136/jitc-2021-003213

Using organoids to identify anti-tumor TCRs from blood

Table S1 (A) Patient information. (B) Total cell number for each patient' opT cells generated. (C) Antibodies and clones used in the CyTOF panel.

Table S2 (A) T cell surface markers to identify naive and memory T cell by CytoF. (B) Detailed sequence information for the top 10 TCRs in opT and TIL for patient 3. (C) The β chain of top 5 TCRs from the opT cells and TlLs were compared with a TCR database from 120 healthy individuals. (D) The percentage (%) of the top 5 TCRs from opT cells were represented in PBMCs from pt3, 10 and 38, respectively.

Table S3 Antibodies used in the flow cytometry for immune checkpoint expression.
