# Supplementary material for: Empirical identification and validation of tumor-targeting T cell receptors from circulation using autologous pancreatic tumor organoids
Source: J Immunother Cancer. 2021 Nov 16;9(11):e003213. doi: 10.1136/jitc-2021-003213 (PMC8601084; doi:10.1136/jitc-2021-003213)
Supplement: Supplementary data [file jitc-2021-003213supp003.pdf]

Table S1

A

Patient ID	Age	Sex	Diagnosis	Histology	Tumor Stage	Degree of apoptosis/necrosis	Degree of Differentiation	IHC based Subtypes	Tumor Grade	Met
Pt3	51	F	PDAC	Adenocarcinoma	4	mild apoptosis, no necrosis	poorly differentiated	Classical	3	Lung
Pt10	67	F	PDAC	Adenocarcinoma	4	no apoptosis, regions of necrosis	well-differentiated	Basal	1	Liver
Pt19	80	F	PDAC	Adenocarcinoma	4	mild apoptosis, regions of necrosis	moderately differentiated	Mixed	2	Liver
Pt38	65	M	PDAC	Adenocarcinoma	4	no apoptosis, no necrosis	moderately differentiated	Mixed	2	Liver, Lung

Patient ID	KRAS _mut	GNAS _mut	CDKN2A/2B _mut	TP53 _mut	SMAD4 _mut	ARID1A _mut	HER2 _mut	DDR gene _mut	Vital Status (months)
Pt3	1	0	1	1	0	0	1	0	35
Pt10	1	0	1	1	0	0	0	CHEK1	22 (alive)
Pt38	1	0	0	1	0	0	0	0	6
Pt19	1	0	1	1	1	0	0	0	NA

B

Patient ID	Start from PBMCs $(\cdot 10^6)$	Final cell number $(\cdot 10^6)$	
		PBMCs (without tumor stimulation)	opT(with tumor stimulaton)
Pt3	0.3	3.3	216.0
Pt10	0.3	0.7	3.4
Pt38	0.3	0.9	4.5
Pt19	0.3	0.5	12.3

C

Antibody	Clone	Isotypes	Source	Cat. No
CD3	UCHT1	115In	BWH CytoF core	V17836
CD4	RPA T4	145Nd	BWH CytoF core	V13137
CD8a	RPA T8	146Nd	BWH CytoF core	V19727
CD19	HIB19	160Gd	BWH CytoF core	V15339
CD56	NCAM16.2	162Dy	BWH CytoF core	V19817
TCR Vα7.2	3C10	169Tm	BWH CytoF core	V20089
CD161	HP-3G10	164Dy	BWH CytoF core	V18502
TCR γδ	B1	173Yb	BWH CytoF core	V19099
iNKT	6B11	163Dy	BWH CytoF core	V13633
CD45RA	HI100	142Nd	BWH CytoF core	V12362
CD45RO	UCHL1	147Sm	BWH CytoF core	V17586
CD197/CCR7	G043H7	159Tb	BWH CytoF core	V15498
CD103	Ber-ACT8	151Eu	BWH CytoF core	V17952
CD27	O323	141Pr	BWH CytoF core	V17009
CD28	CD28.2	148Nd	BWH CytoF core	V19038
CD62L/L-Selectin	DREG-56	174Yb	BWH CytoF core	V16535
CD95/FAS	DX2	156Gd	BWH CytoF core	V14020
CD117/C-kit	104D2	158Gd	BWH CytoF core	V18238
CD314/NKG2D	5C6	166Er	BWH CytoF core	V10008
CD154/CD40L	24-31	154Sm	BWH CytoF core	V18377
CD25	M-A251	149Sm	BWH CytoF core	V17042
CD44	IM7	113In	BWH CytoF core	V16308
CD69	FN50	153Eu	BWH CytoF core	V19261
CD279/PD1	EH12.2H7	143Nd	BWH CytoF core	V17017
CD152/CTLA-4	L3D10	152Sm	BWH CytoF core	V16490
CD196/CCR6	G034E3	168Er	BWH CytoF core	V18535
CD194/CCR4	L291H4	150Nd	BWH CytoF core	V15278
CXCR3	G025H7	161Dy	BWH CytoF core	V20053
