# Supplementary material for: Empirical identification and validation of tumor-targeting T cell receptors from circulation using autologous pancreatic tumor organoids
Source: J Immunother Cancer. 2021 Nov 16;9(11):e003213. doi: 10.1136/jitc-2021-003213 (PMC8601084; doi:10.1136/jitc-2021-003213)
Supplement: Supplementary data [file jitc-2021-003213supp005.pdf]

Table S3

Antibody	Color	Clone	Company	Catalogue No.
CD3	PE	HIT3a	Biolegend	300308
CD3	BV605	UCHT1	Biolegend	300460
CD4	APC	OKT4	Biolegend	317416
CD4	BV650	OKT4	Biolegend	317435
CD8	FITC	SK1	BD	347313
CD8	PerCP	SK1	Biolegend	344707
LAG-3 (CD223)	BV 605	11C3C65	Biolegend	369323
TIM-3 (CD366)	APC	F38-2E2	Biolegend	345011
TIGIT (VSTM3)	APC	A15153G	Biolegend	372705
BTLA (CD272)	PE	MIH26	Biolegend	344505
GITR (CD357)	PerCP/Cyanine5.5	108-17	Biolegend	371217
CD96	PE	NK92.39	Biolegend	338405
NKG2A	BV711	131411 (RUO)	BD	747919
KIR2DL1 (CD158a)	PE	REA284	Miltenyi Biotec	130-120-586
KIR2DL2/DL3 (CD158b)	PerCP	DX27	Miltenyi Biotec	130-099-700
KIR2DL4 (CD158d)	FITC	REA768	Miltenyi Biotec	130-112-536
KIR2DL5 (CD158f)	PE	REA955	Miltenyi Biotec	130-115-911
KIR3DL1/DL2(CD158e/k)	FITC	REA970	Miltenyi Biotec	130-116-280
KIR3DL3 (CD158z)	AF647	1136B	R&D systems	FAB8919R-025
