# Supplementary figures and images for: Empirical identification and validation of tumor-targeting T cell receptors from circulation using autologous pancreatic tumor organoids
Source: J Immunother Cancer. 2021 Nov 16;9(11):e003213. doi: 10.1136/jitc-2021-003213 (PMC8601084; doi:10.1136/jitc-2021-003213)

Fig. S1

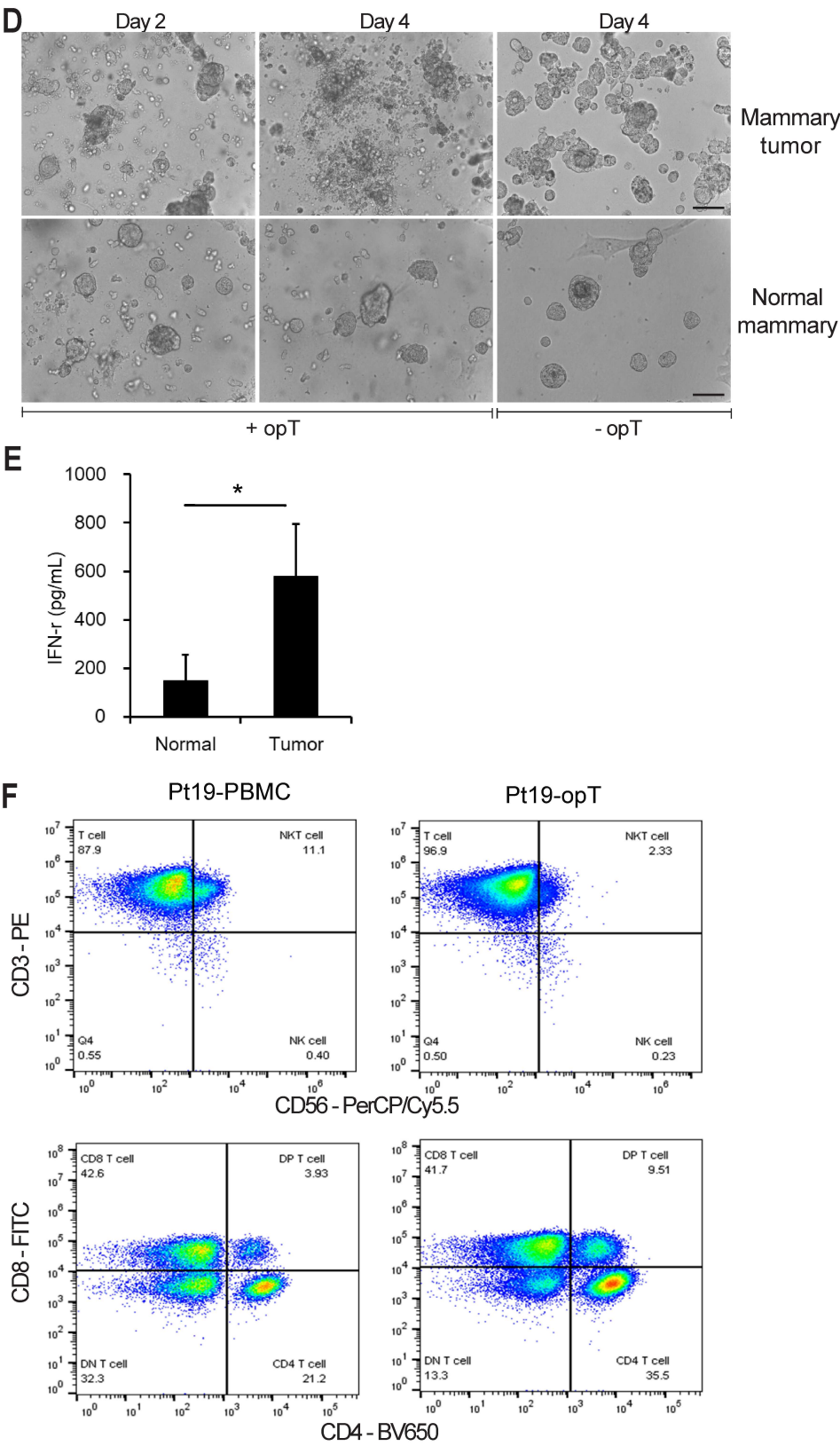

Supplement: Supplementary data [file jitc-2021-003213supp006.pdf]

Fig. S1

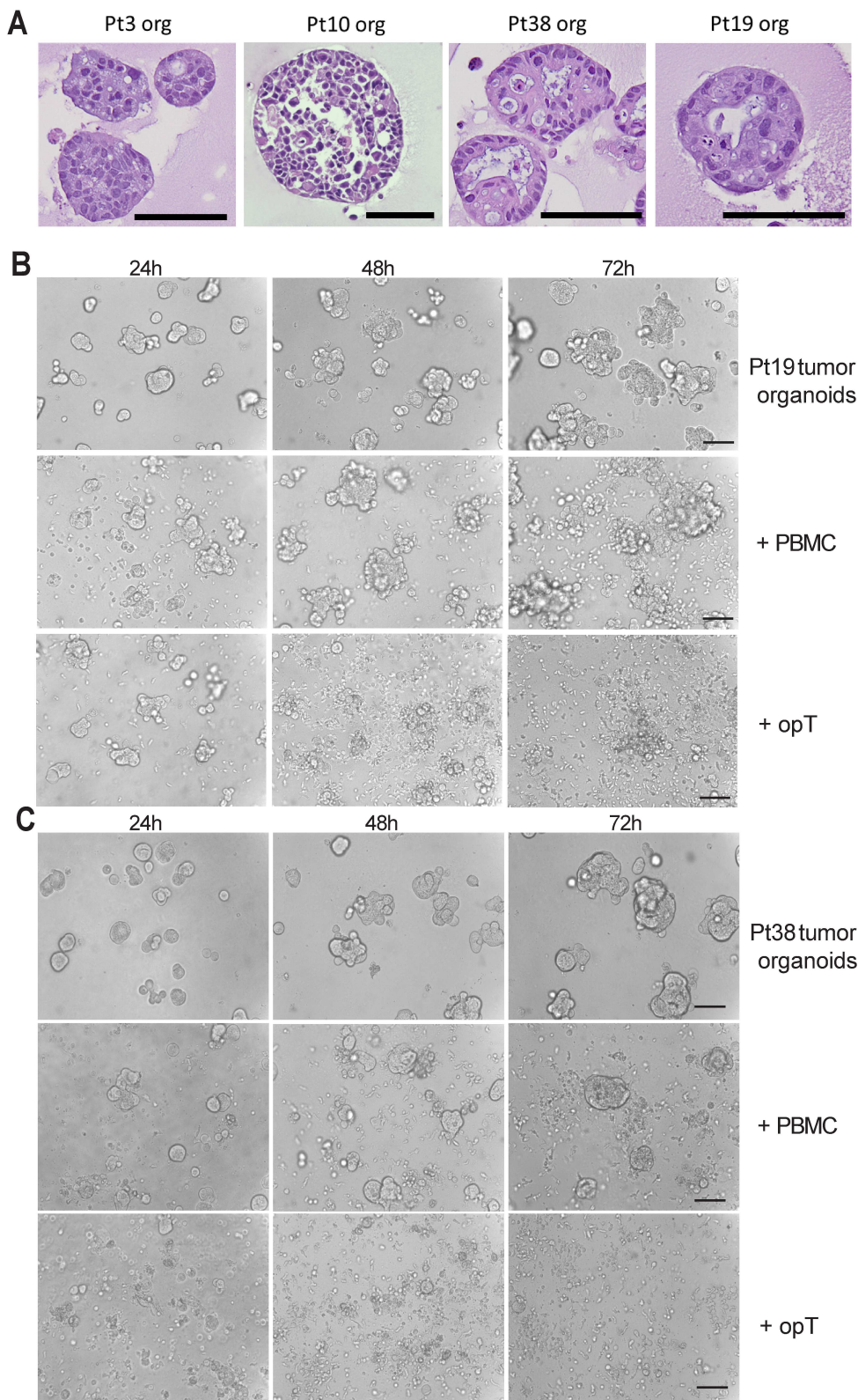

Supplement: Supplementary data [file jitc-2021-003213supp007.pdf]

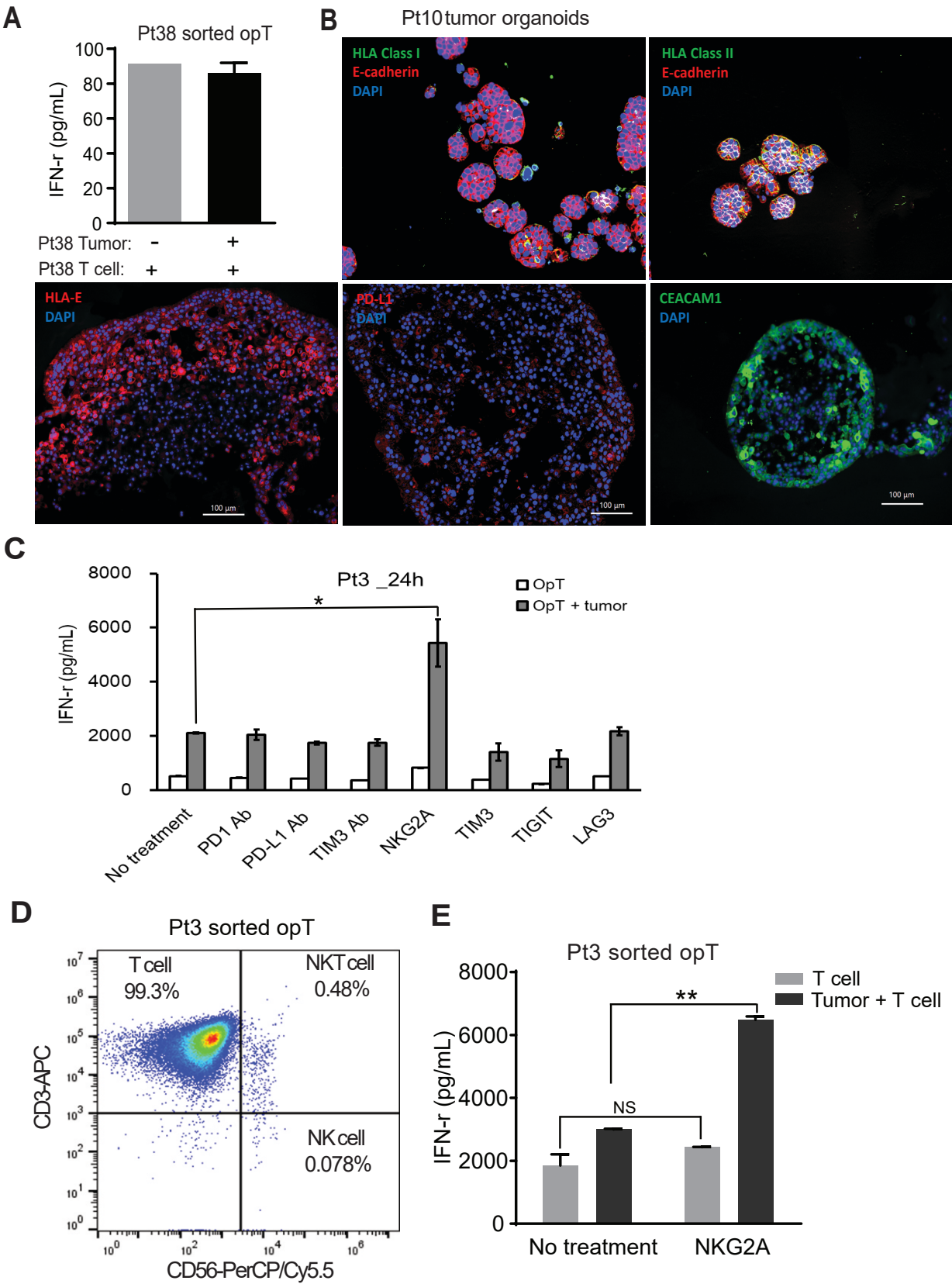

Supplement: Supplementary data [file jitc-2021-003213supp008.pdf]

Fig. S3

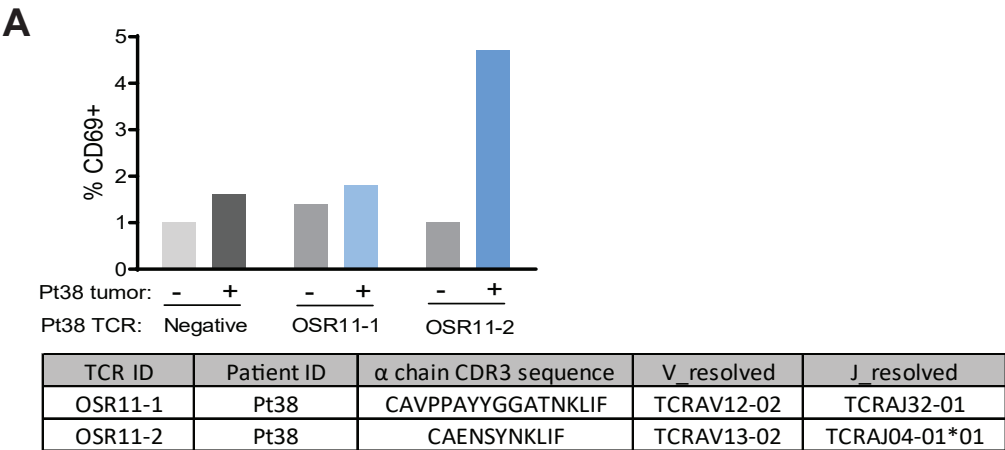

Supplement: Supplementary data [file jitc-2021-003213supp009.pdf]
