# Supplementary material for: Empirical identification and validation of tumor-targeting T cell receptors from circulation using autologous pancreatic tumor organoids
Source: J Immunother Cancer. 2021 Nov 16;9(11):e003213. doi: 10.1136/jitc-2021-003213 (PMC8601084; doi:10.1136/jitc-2021-003213)
Supplement: Supplementary data [file jitc-2021-003213supp004.pdf]

Table S2

A

Phenotype Key	
T cell	CD3+
CD4+ or CD8+	
Tissue-resident memory (TRM)	CD69+,CD103-,CCR7-,CD45RO+
CD103+ TRM	CD69+,CD103+,CCR7-,CD45RO+
Naive	CD69-,CD103-,CD95-,CD28+,CCR7+,CD45RO-
Stem Cell Memory	CD69-,CD103-,CD95+,CD28+,CCR7+,CD45RO-
Central memory	CD69-,CD103-,CD95+,CD28+,CCR7+,CD45RO+
Transitional memory	CD69-,CD103-,CD95+,CD28+,CCR7-,CD45RO+
Effector memory	CD69-,CD103-,CD95+,CD28-,CCR7-,CD45RO+
Terminal effector	CD69-,CD103-,CD95+,CD28-,CCR7-,CD45RO-

B

	TCR Rank	Frequency (%)	β chain CDR3 sequence	V_resolved	D_resolved	J_resolved
Pt3 opT	1	81.0	CASSSRDRMNTEAFF	TCRBV06-05*01	TCRBD01-01*01	TCRBJ01-01*01
	2	9.3	CASSLWSTPTDTQYF	TCRBV07-03*01	TCRBD02-01	TCRBJ02-03*01
	3	5.9	CASSANRAPNEKLFF	TCRBV27-01*01	TCRBD01-01*01	TCRBJ01-04*01
	4	1.6	CASSPTIEETQYF	TCRBV06-01*01	TCRBD02-01	TCRBJ02-05*01
	5	1.3	CASRSSNQPQHF	TCRBV19-01*01	TCRBD02-01	TCRBJ01-05*01
	6	0.20	CASSLAGEVEDTQYF	TCRBV07-07*01	TCRBD02-01*02	TCRBJ02-03*01
	7	0.13	CASSTGDEQFF	TCRBV02-01	TCRBD01-01*01	TCRBJ02-01*01
	8	0.11	CASSEQRGGFNEQFF	TCRBV02-01	TCRBD02-01*01	TCRBJ02-01*01
	9	0.04	CASSTTLRDRGLANTGELFF	TCRBV28-01*01	TCRBD01-01*01	TCRBJ02-02*01
	10	0.03	CASSYSDSGKIYYEQYF	TCRBV06-07*01	TCRBD02-01*02	TCRBJ02-07*01
Pt3 TIL	1	50.8	CASSPTIEETQYF	TCRBV06-01*01	TCRBD02-01	TCRBJ02-05*01
	2	30.5	CASSQDGIAGETQYF	TCRBV04-01*01	TCRBD02-01*01	TCRBJ02-05*01
	3	14.1	CSAADPYSYEQYF	TCRBV20	TCRBD02-01	TCRBJ02-07*01
	4	2.8	CASSGDSSYGYTF	TCRBV12-03/12-04*01	TCRBD01-01*01	TCRBJ01-02*01
	5	1.3	CASRPSRATNEKLFF	TCRBV27-01*01	unknown	TCRBJ01-04*01
	6	0.28	CATSRDQGLASNEQFF	TCRBV15-01*02	TCRBD02-01	TCRBJ02-01*01
	7	0.04	CASSEDRRYGYTF	TCRBV07-02	TCRBD01-01*01	TCRBJ01-02*01
	8	0.01	CAWSVRGLPQHF	TCRBV30-01*01	TCRBD02-01*01	TCRBJ01-05*01
	9#	0.01	CASSRVGLDYEQYF	TCRBV21-01*01	TCRBD02-01	TCRBJ02-07*01
	10	0.01	CASRSSNQPQHF	TCRBV19-01*01	TCRBD02-01	TCRBJ01-05*01

(Same background color: overlapped TCRs between Pt3 opT and TIL; #: overlapped with rank 52 TCR in Pt3 opT)

C

	TCR ID	TCR Rank	Percentage (%) in		
			Pt3 PBMC	Pt10 PBMC	Pt38 PBMC
Pt3 opT	OSR 1	1	2.3	—	—
	OSR 2	2	0.7	—	—
	OSR 3	3	0.5	—	—
	OSR 4	4	8.6	—	—
	OSR 5	5	2.4	—	—
Pt10 opT	OSR 6	1	—	0.2	—
	OSR 7	2	—	0.7	—
	OSR 8	3	—	—	—
	OSR 9	4	—	2.6	—
	OSR 10	5	—	—	—
Pt38 opT	OSR 11	1	—	—	—
	OSR 12	2	—	—	1.7
	OSR 13	3	—	—	0.5
	OSR 14	4	—	—	0.5
	OSR 15	5	—	—	2.0

(— , undetected.)

D

	TCR ID	TCR Rank	No. of healthy controls with the same TCR in total 120	Highest TCR frequency
Pt3 opT	OSR 1	1	0	—
	OSR 2	2	1	3.00E-06
	OSR 3	3	0	—
	OSR 4	4	0	—
	OSR 5	5	6	4.3569E-06
Pt10 opT	OSR 6	1	0	—
	OSR 7	2	0	—
	OSR 8	3	0	—
	OSR 9	4	0	—
	OSR 10	5	0	—
Pt38 opT	OSR 11	1	25	1.15821E-05
	OSR 12	2	8	7.2296E-06
	OSR 13	3	0	—
	OSR 14	4	0	—
	OSR 15	5	0	—
Pt3 TIL	TIR 1	1	0	—
	TIR 2	2	0	—
	TIR 3	3	2	6.1257E-06
	TIR 4	4	0	—
	TIR 5	5	0	—

(— , undetected.)
